# Effects of peripheral blood leukocyte count and tumor necrosis factor-alpha on early death in acute promyelocytic leukemia

**DOI:** 10.1186/s12885-022-10499-2

**Published:** 2023-01-07

**Authors:** Jingjing Wen, Fang Xu, Qiaolin Zhou, Lin Shi, Yiping Liu, Jing Yue, Ya Zhang, Xiaogong Liang

**Affiliations:** Department of Hematology, Mianyang Central Hospital, School of Medicine, University of Electronic Science and Technology of China, No 12. Changjia alley, Jingzhong Street, Fucheng district, Mianyang, 621000 China

**Keywords:** Leukemia, promyelocytic, acute, Leukocyte count, Tumor necrosis factor-alpha, Cell differentiation, Mortality, Prognosis

## Abstract

**Background:**

Early death remains a major factor in survival in APL. We aimed to analyze the risk factors for differentiation syndrome and early death in acute promyelocytic leukemia (APL).

**Methods:**

The clinical data of APL patients who were newly diagnosed at Mianyang Central Hospital from January 2013 to January 2022 were retrospectively analyzed.

**Results:**

Eighty-six newly diagnosed APL patients (37 males and 49 females) were included in this study. The median age was 46 (17–75) years. Sixty-one patients (70.9%) had low/intermediate-risk APL, and 25 patients (29.1%) had high-risk APL. The incidence of differentiation syndrome (DS) was 62.4%. The multivariate analysis showed that a peak white blood cell (WBC) count ≥16 × 10^9/L was an independent risk factor (OR = 11.000, 95% CI: 2.830–42.756, *P* = 0.001) for DS in all APL patients, while a WBC count ≥10 × 10^9/L on Day 5 was an independent risk factor for DS in low-intermediate risk APL patients (OR = 9.114, 95% CI: 2.384–34.849, *P* = 0.001). There were 31 patients (36.5%) with mild DS and 22 patients (25.9%) with severe DS. The multivariate analysis showed that WBC count ≥23 × 10^9/L at chemotherapy was an independent risk factor for severe DS (OR = 10.500, 95% CI: 2.344–47.034, *P* = 0.002). The rate of early death (ED) was 24.4% (21/86). The multivariate analysis showed that male gender (OR = 7.578,95% CI:1.136–50.551, *P* = 0.036), HGB < 65 g/L (OR = 16.271,95% CI:2.012–131.594, *P* = 0.009) and WBC count ≥7 × 10^9/L on Day 3(OR = 23.359,95% CI:1.825–298.959, *P* = 0.015) were independent risk factors for ED. The WBC count at diagnosis, WBC count on Day 3 and WBC count on Day 5 had moderate positive correlations with tumor necrosis factor-α (TNF-α) at diagnosis, and the correlation coefficients were 0.648 (*P* = 0.012), 0.615 (*P* = 0.033), and 0.609 (*P* = 0.035), respectively. The WBC count had no correlation with IL-6.

**Conclusion:**

During induction treatment, cytotoxic chemotherapy may need to be initiated to reduce the risk of DS for APL patients with a low-intermediate risk WBC count ≥10 × 10^9/L on Day 5 or for all patients with a peak WBC count ≥16 × 10^9/L. Patients with WBC > 7 × 10^9/L on Day 3 have a higher risk of ED. Leukocyte proliferation is associated with TNF-α rather than IL-6, and TNF-α may be a potential biomarker for predicting ED.

**Supplementary Information:**

The online version contains supplementary material available at 10.1186/s12885-022-10499-2.

## Introduction

Acute promyelocytic leukemia (APL) has become a highly curable hematologic neoplastic disease with a 10-year overall survival (OS) rate of 93.9% [1] due to the use of all-trans retinoic acid (ATRA) and arsenic trioxide (ATO). With increasingly effective treatments, early mortality can still be as high as 32.6–34.6% [2–4]. Early death remains a major factor of survival in APL. Strategies to reduce early death are exceedingly important for improving survival in APL [3, 5]. Differentiation syndrome (DS) remains one of the most frequently cited causes of early mortality [6]. To better prevent early death, patients with a high risk of early death and DS should be categorized or identified. We performed a study of 86 APL patients who were newly diagnosed at Mianyang Central Hospital from January 2013 to January 2022 to explore the risk factors for differentiation syndrome and early death.

## Patients and methods

### Patients

This is a retrospective study in a single institution. The clinical data of all newly diagnosed APL patients hospitalized in the Hematology Department of Mianyang Central Hospital from January 2013 to January 2022 were collected. The diagnosis was in accordance with the Chinese guidelines for the diagnosis and treatment of APL (2018) [7]. All enrolled patients were newly diagnosed and aged ≥16 years old. They were positive for the PML/RARα gene. The exclusion criteria included patients with secondary leukemia or who had disease combined with secondary malignancies, as well as pregnant or lactating women. The medical records of all patients were reviewed in detail to obtain information regarding clinical characteristics, laboratory parameters, treatment, efficacy, early death and survival. The patient laboratory data included complete blood count, coagulation, lactate dehydrogenase (LDH) and renal function at diagnosis and during induction treatment. The levels of tumor necrosis factor-α (TNF-α) and interleukin-6 (IL-6) were tested by chemiluminescence (Siemens immulite 1000). The bone marrow-related examinations included the percentage of bone marrow promyelocytes, immunophenotype by flow cytometry, and PML-RARα genotyping. All patients signed written informed consent.

### Treatment regimens and evaluation

All patients with suspected APL immediately received oral ATRA (25 mg/m^2^/day). After the diagnosis of APL was established, ATO (0.16 mg/kg/day) combined with ATRA was administered as induction therapy. Chemotherapy (hydroxyurea, anthracyclines, cytarabine and homoharringtonine) was administered when the white blood cell (WBC) count was higher than 4 × 10^9/L during induction therapy and was decided by the clinician. The dosage protocol for all combined chemotherapy agents was given according to the Chinese Diagnosis and Treatment Guidelines for APL (2011, 2014, 2018 version), which was as follows: daunorubicin 25–45 mg/m^2^/day on Days 1 ~ 3, idarubicin 8–12 mg/m^2^/day on Days 1 ~ 3, cytarabine 100 mg/m^2^/day on Days 1 ~ 5 or 7, homoharringtonine 1–2 mg/m^2^/day on Days 1 ~ 5 or 7, and hydroxyurea 3.0 g/day. We adjusted the dosages according to the WBC count, patient tolerance and complications. The diagnosis of remission and relapse was based on the literature [8].

### Definition of end points

Prognostic stratification was performed according to the Sanz criteria [9], with a WBC count ≤10 × 10^9/L defined as low-intermediate risk and a WBC count > 10 × 10^9/L defined as high risk. Differentiation syndrome (DS) was diagnosed according to the definition of Frankel [10] and in patients with the following symptoms and signs: respiratory distress, unexplained fever, weight gain > 5 kg, pleural effusion, pericardial effusion, hypotension and acute renal failure. DS was classified as either mild or severe according to the literature [11]. If there were 4 or more symptoms, the patient was classified with severe DS. If there were 2 or 3 symptoms, DS was classified as mild DS. Early death was defined as death within the first 30 days of presentation. OS was defined as the time from diagnosis to death (event) or last follow-up. Follow-up was performed by telephone and outpatient medical records. The optimal threshold was derived from the ROC curve. The follow-up deadline was February 28, 2022, with a median follow-up of 28.0 (0–111.0) months. Infection and hemorrhage were defined according to the Common Terminology Criteria for Adverse Events (CTCAE) version 5.0.

### Statistical analysis

All statistical analyses were performed with SPSS version 26.0. Measurement data conforming to a normal distribution are represented by^−^χ ± s. Nonnormal measurement data are expressed as the median (range). Categorical variables were analyzed by the chi-square or Fisher exact test, and continuous variables were analyzed by the t test or nonparametric test if the data was not normally distributed. Multivariate analysis was carried out by a logistic regression model. OS was analyzed by the Kaplan–Meier method, and the difference between the groups was determined by the log-rank test. Spearman correlation was used for correlation analysis. A two-tailed *P* < 0.05 was regarded as significant. The figures were drawn by GraphPad Prism 8.0.

## Results

### Patient characteristics

Eighty-six newly diagnosed APL patients (37 males and 49 females) were included in this study. The median age was 46 (17–75) years, and 11 patients (12.8%) were over 60 years old. The median age of the male patients was 43.07 (18–75) years, and the median age of the female patients was 47.02 (17–71), *P* = 0.403. Sixty-one patients (70.9%) had low/intermediate-risk APL, and 25 patients (29.1%) had high-risk APL. At the time of diagnosis or during therapy, 89.5% (77/86) of the patients suffered from infection. Hemorrhage occurred in 87.2% (75/86) of the patients. The main bleeding sites included the skin (40%, 30/75), the oral mucous membranes (25.3%, 19/75), and intracranial (12%, 9/75). A total of 49.3% (37/75) of the patients had only one bleeding site, 34.7% (26/75) had two bleeding sites, and 16.0% (12/75) had three bleeding sites. At diagnosis, the median WBC count was 3.95 (0.19–192.63) × 10^9/L, the median hemoglobin (HGB) level was 79 (33–128) g/L g/L, the median PLT count was 19 (4–95) × 10^9/L, the median fibrinogen (FIB) level was 1.28 (0.24–5.35) g/L, and the median D-dimer level was 9.84 (0.75–40.10) mg/L FEU. The median time to achieve remission in the bone marrow was 24.5 (17–41) days.

A total of 18.6% (16/86) of the patients did not receive cytotoxic agent-based chemotherapy. The patients who were not treated with combination chemotherapy were those with low/intermediate risk. A total of 33.7% (29/86) of the patients received combination chemotherapy 3 days after ATRA induction treatment. A total of 45.3% (39/86) received combination chemotherapy in 5 days. A total of 45 patients with low/intermediate risk received cytoreduction chemotherapy. Eighteen patients received daunorubicin or idarubicin alone, and 4 patients received daunorubicin or idarubicin combined with cytarabine. Four patients received homoharringtonine alone, and 8 patients received homoharringtonine combined with cytarabine. The other 11 patients only received hydroxyurea. In the high-risk group, 13 patients received daunorubicin or idarubicin alone, 3 patients received homoharringtonine alone, 3 patients received homoharringtonine combined with cytarabine, and 6 patients received cytarabine alone. Chemotherapy was added in 73.8% of the low/intermediate-risk APL patients. All 25 high-risk APL patients received combination chemotherapy. Long-term survival was achieved in the patients without early death (Supplementary Table).

Male patients accounted for 76.2% (16/21) of the patients with early deaths, among which 13 patients were younger than 60 years and 3 patients were older than 60 years. The early death rates were higher for males than females in both the younger (< 60 years) and older (≥60 years) patients. (Table 1).

**Table 1:** Clinical characteristics in patients with acute promyelocytic leukemia at diagnosis by gender and age

Clinical characteristics	Age		Gender		Age < 60 years		Age ≥ 60 years	
	< 60 years (*n* = 75)	≥60 years (*n* = 11)	Male (*n* = 37)	Female (*n* = 49)	Male	Female	Male	Female
Gender								
Male	89.2%(33/37)	10.8%(4/37)	–	–	–	–	–	–
Female	85.7%(42/49)	14.3%(7/49)	–	–	–	–	–	–
WBC count at diagnosis (× 10^9^/L)	4.24 (0.19 ~ 192.63)	1.2 (0.52 ~ 30.30)	4.24 (0.19 ~ 192.63)	3.39 (0.42 ~ 97.22)	4.26 (0.19 ~ 192.63)	3.57 (0.42 ~ 97.22)	1.02 (0.84 ~ 4.26)	2.02 (0.52 ~ 30.30)
PLT count at diagnosis (×10^9^/L)	21(4 ~ 95)	17(4 ~ 57)	21(4 ~ 75)	18(4 ~ 95)	21.0(4 ~ 75)	19(4 ~ 95)	8(4 ~ 26)	18(4 ~ 75)
Risk group								
Low/intermediate	83.6%(51/61)	16.4%(10/61)	42.6%(26/61)	57.4%(35/61)	43.1%(22/51)	56.9%(29/51)	40.0%(4/10)	60.0%(6/10)
High	96.0%(24/25)	4%(1/25)	44.0%(11/25)	56.0%(14/25)	45.8%(11/24)	54.2%(13/24)	0	100%(1/1)
Occurrence of DS								
Yes	88.7%(47/53)	11.3%(6/53)	39.6%(21/53)	60.4%(32/53)	40.4%(19/47)	59.6%(28/47)	33.3%(2/6)	66.7%(4/6)
No	84.4%(27/32)	15.6%(5/32)	46.9%(15/32)	53.1%(17/32)	48.1%(13/27)	51.9%(14/27)	40.0%(2/5)	60.0%(3/5)
Severity of DS								
mild	90.3%(28/31)	9.7%(3/31)	38.7%(12/31)	61.3%(19/31)	39.3%(11/28)	60.7%(17/28)	33.3%(1/3)	66.7%(2/3)
severe	86.4%(19/22)	13.6%(3/22)	40.9%(9/22)	59.1%(13/22)	42.1%(8/19)	57.9%(11/19)	33.3%(1/3)	66.7%(2/3)
Early death								
Yes	85.7%(18/21)	14.3%(3/21)	76.2%(16/21)^*^	23.8%(5/21)^*^	72.2%(13/18) ^*^	27.8%(5/18) ^*^	100%(3/3) ^*^	0^*^
No	87.7%(57/65)	12.3%(8/65)	32.3%(21/65)^*^	67.7%(44/65)^*^	35.1%(20/57) ^*^	64.9%(37/57) ^*^	12.5%(1/8) ^*^	87.5%(7/8) ^*^

*DS* differentiation syndrome, *WBC* white blood cell, *PLT* platelet

^*^P < 0.05

### Differentiation syndrome

DS occurred in 53 patients (62.4%), 32 patients (37.5%) without DS, and whether one patient developed DS was unknown. The median time to DS was 8 (3–19) days. The WBC counts (at diagnosis, Day 5, peak) in the patients with DS were significantly higher than those in the patients without DS (all *P* < 0.05, Table 2). The univariate analysis showed that the proportion of DS patients with WBC count ≥10 × 10^9/L on the 5th day was significantly higher than that of the patients with WBC count < 10 × 10^9/L on the 5th day, and the difference was statistically significant (*P* < 0.05). The proportion of bone marrow promyelocytes and the amount of blood transfusion in the patients with DS were significantly higher than those in the patients without DS (all P < 0.05, Table 2). The patients with DS had a slightly longer time to achieve remission than those without DS, but the difference was not statistically significant (*P* = 0.067).

**Table 2:** Univariate analysis of differentiation syndrome in patients with acute promyelocytic leukemia

Clinical characteristics	Patients without DS(*n* = 32cases)	Patients with DS(*n* = 53 cases)	Statistical value (χor Z value)	*P*-value
WBC count at diagnosis (× 10^9^/L)	1.77(0.19 ~ 192.63)	4.92(0.46 ~ 108.75)	1.968	0.049
WBC count at diagnosis≥3 × 10^9^/L	26.1%(12/46)	73.9%(34/46)	5.708	0.017
WBC count at diagnosis< 3 × 10^9^/L	51.3%(20/39)	48.7%(19/39)		
Percentage of monocytes at diagnosis (%)	23.15(2.3 ~ 88.4)	41.8(0.9 ~ 85.9)	2.037	0.042
WBC on Day 5				
WBC count on Day 5 ≥ 10 × 10^9^/L	20.5%(8/39)	79.5%(31/39)	5.341	0.021
WBC count on Day 5 < 10 × 10^9^/L	46.7%(14/30)	53.3%(16/30)		
Peak WBC (×10^9^/L)	18.26(1.56 ~ 194.43)	32.70(4.35 ~ 112.85)	−2.807	0.005
Peak WBC count ≥16 × 10^9^/L	22.2%(12/54)	77.8%(42/54)	12.112	0.001
Peak WBC count < 16 × 10^9^/L	68.8%(11/16)	31.3%(5/16)		
Percentage of bone marrow promyelocytes (%)	77.5(5.0 ~ 96.5)	85.0(26.0 ~ 95.5)	−2.039	0.041
Time for bone marrow to achieve remission(day)	22(17 ~ 35)	26(17 ~ 41)	−1.835	0.067
Transfusion of RBC (U)	5.0(0 ~ 15)	8.0(0 ~ 22)	−3.277	0.001
Transfusion of PLT (therapeutic volumes)	1(0 ~ 6)	2(0 ~ 12)	−3.769	0.000

*DS* differentiation syndrome, *WBC* white blood cells, *RBC* red blood cells, *PLT* platelets

In all patients, multivariate analysis was used to examine the effects of WBC count ≥3 × 10^9/L at diagnosis, peak WBC count ≥16 × 10^9/L, percentage of monocytes at diagnosis, percentage of bone marrow promyelocytes and WBC count ≥10 × 10^9/L at Day 5 on DS. A peak WBC count ≥16 × 10^9/L was an independent risk factor for DS in APL (OR = 11.000, 95% CI: 2.830–42.756, *P* = 0.001). In the patients with low-intermediate risk APL, WBC count ≥3 × 10^9/L, peak WBC count ≥16 × 10^9/L, percentage of monocytes at diagnosis and WBC count ≥10 × 10^9/L on Day 5 were included in the multivariate analysis. WBC count ≥10 × 10^9/L on Day 5 was found to be an independent risk factor for DS (OR = 9.114, 95% CI: 2.384–34.849, *P* = 0.001).

### Severity of DS

Thirty-one patients (36.5%) suffered from mild DS, and 22 patients (25.9%) had severe DS. The D-dimer levels were elevated in all patients with DS, and the median D-dimer level was significantly higher in the severe DS group than in the mild DS group. The univariate analysis showed that the incidence of severe DS was significantly increased when the D-dimer level was ≥12 mg/L FEU (0–0.55 mg/L FEU). The WBC counts on Day 3 and at chemotherapy were both significantly higher in the patients with severe DS than in those with mild DS. According to the cutoff value of the ROC curve, the univariate analysis showed that the proportion of patients with a WBC count ≥16 × 10^9/L on Day 3 was significantly higher than that of the patients with a WBC count < 16 × 10^9/L on Day 3. The proportion of patients with WBC counts ≥23 × 10^9/L at chemotherapy was significantly higher than the proportion of patients with WBC counts < 23 × 10^9/L at chemotherapy. The proportion of patients with elevated LDH and the amount of blood transfusion in the severe DS group were significantly higher than those in the mild DS group. All the above differences were statistically significant (*P* < 0.05, Table 3).

**Table 3:** Univariate analysis of the severity of differentiation syndrome in patients with acute promyelocytic leukaemia

Clinical characteristics	mild DS	severe DS	Statistical value (χorZ value)	*P*-value
Transfusion of RBC (U)	6.5(0 ~ 16)	10.0(2 ~ 22)	−2.323	0.020
Transfusion of PLT (therapeutic volumes)	2.0(0 ~ 7)	4.0(0 ~ 12)	−2.504	0.012
D-dimer (mg/L FEU)	7.34(0.75 ~ 17.15)	14.77(0.77 ~ 40.10)	−2.177	0.030
D-dimer≥12 mg/L FEU	11.1%(1/9)	88.9%(8/9)	5.091	0.024
D-dimer< 12 mg/L FEU	69.2%(9/13)	30.8%(4/13)		
WBC count on Day 3 (×10^9^/L)	7.24(0.63 ~ 60.96)	16.46(0.56 ~ 69.58)	−2.013	0.044
WBC count on Day 3 ≥ 16 × 10^9^/L	35.3%(6/17)	64.7%(11/17)	6.947	0.008
WBC count on Day 3 < 16 × 10^9^/L	74.2%(23/31)	25.8%(8/31)		
WBC count at chemotherapy (×10^9^/L)	19.78(7.77 ~ 73.05)	54.62(6.24 ~ 113.20)	−3.394	0.001
WBC count at chemotherapy≥23 × 10^9^/L	36.0%(9/25)	64.0%(16/25)	13.006	0.000
WBC count at chemotherapy< 23 × 10^9^/L	87.0%(20/23)	13.0%(3/23)		
LDH > 250 U/L	60.0%(18/30)	86.4%(19/22)	4.298	0.038

*DS* differentiation syndrome, *WBC* white blood cells, *RBC* red blood cells, *PLT* platelets, *LDH* lactate dehydrogenase, D-dimer reference range is 0–0.55 mg/L FEU

A multivariate analysis was used to examine the effects of WBC count ≥16 × 10^9/L on Day 3, WBC count ≥23 × 10^9/L at chemotherapy and LDH > 250 U/L on the severity of DS. WBC count ≥23 × 10^9/L at chemotherapy was an independent risk factor for severe DS (OR = 10.500, 95% CI: 2.344–47.034, *P* = 0.002).

### Early death and survival analysis

The early death rate was 24.4% (21/86). Compared with the survival group, the early death group had more men, a lower incidence of DS, a higher proportion of severe DS, a higher proportion of HGB < 65 g/L at diagnosis, a higher proportion of GFR < 60 ml/min, higher cystatin C levels and higher CRP levels, and the above differences were statistically significant (all *P* < 0.05). The median WBC counts on Days 3 and 5 were significantly higher in the early death group than in the survival group. According to the ROC curve, by taking a WBC count of 7 × 10^9/L on Day 3 as the cutoff value, we found that the early mortality of the patients with a WBC count ≥7 × 10^9/L on Day 3 was significantly higher than that of patients with a WBC count < 7 × 10^9/L on Day 3 (Table 4). There was no significant difference between the early death group and the survival group in the WBC counts at diagnosis (*P* = 0.924) and at chemotherapy (*P* = 0.056).

**Table 4:** Univariate analysis of early death in patients with acute promyelocytic leukaemia

Clinical characteristics	Early death group (*n* = 21cases)	Survival group (*n* = 65 cases)	Statistical value (χor t or Z value)	*P*-value
Gender			12.469	0.000
male	76.2%(16/21)	32.3%(21/65)		
female	23.8%(5/21)	67.7%(44/65)		
Occurrence of DS			6.991	0.008
Yes	38.1%(8/21)	70.3%(45/64)		
No	61.9%(13/21)	29.7%(19/64)		
Severity of DS			6.129	0.013
mild	12.5%(1/8)	66.7%(30/45)		
severe	87.5%(7/8)	33.3%(15/45)		
HGB at diagnosis			4.616	0.032
≥ 65 g/L	57.1%(12/21)	83.1%(54/65)		
< 65 g/L	42.9%(9/21)	16.9%(11/65)		
GFR < 60 ml/min	20%(4/20)	9.8%(6/61)	4.100	0.043
Creatinine > Upper limit^a^ (mmol/L)	29.4%(5/17)	7.1%(4/56)	0.652	0.419
LDH > 250 U/L	85%(17/20)	61.3%(38/62)	3.849	0.05
WBC count on Day 3 (×10^9^/L)	19.35(1 ~ 249.98)	5.9(0.43 ~ 71.98)	−2.705	0.007
WBC count on Day 3 ≥ 7 × 10^9^/L	27.5%(11/40)	72.5%(29/40)	7.605	0.006
WBC count on Day 3 < 7 × 10^9^/L	3.1%(1/32)	96.9%(31/32)		
WBC count on Day 5 (×10^9^/L)	20.54(2.17 ~ 250.32)	10.43(0.24 ~ 112.85)	−2.138	0.033
Cystatin C at diagnosis (mg/L)	1.07(0.81 ~ 2.07)	0.92(0.45 ~ 1.63)	−2.258	0.024
Cystatin C on Day 3 (mg/L)	1.71(0.94 ~ 5.91)	0.97(0.5 ~ 1.53)	−2.318	0.02
Cystatin C on Day 5 (mg/L)	3.33(0.97 ~ 5.09)	1.005(0.6 ~ 1.64)	−2.337	0.019
Cystatin C at chemotherapy (mg/L)	3.245(1.04 ~ 5.55)	0.97(0.7 ~ 2.29)	−2.476	0.013
CRP on Day 5 (mg/L)	119.71(106.08 ~ 133.33)	11.47(1 ~ 104.58)	−2.258	0.024
CRP at chemotherapy (mg/L)	187.71(32.65 ~ 320)	24.06(1.41 ~ 87.82)	−2.378	0.017

*DS* differentiation syndrome, *HGB* hemoglobin, *GFR* glomerular filtration rate, *WBC* White blood cells, *LDH* Lactate dehydrogenase, *CRP* C-reactive protein

^a^Upper limit of creatinine reference value (97 mmol/L for male and 73 mmol/L for female)

A multivariate analysis was performed and included sex, DS, HGB < 65 g/L, GFR < 60 ml/min, and WBC count ≥7 × 10^9/L on Day 3. We found that male sex (OR = 7.578,95% CI:1.136–50.551, *P* = 0.036), HGB < 65 g/L (OR = 16.271,95% CI:2.012–131.594), *P* = 0.009) and WBC count ≥7 × 10^9/L on Day 3 (OR = 23.359,95% CI:1.825–298.959, *P* = 0.015) were independent risk factors for early death.

**Fig. 1:**
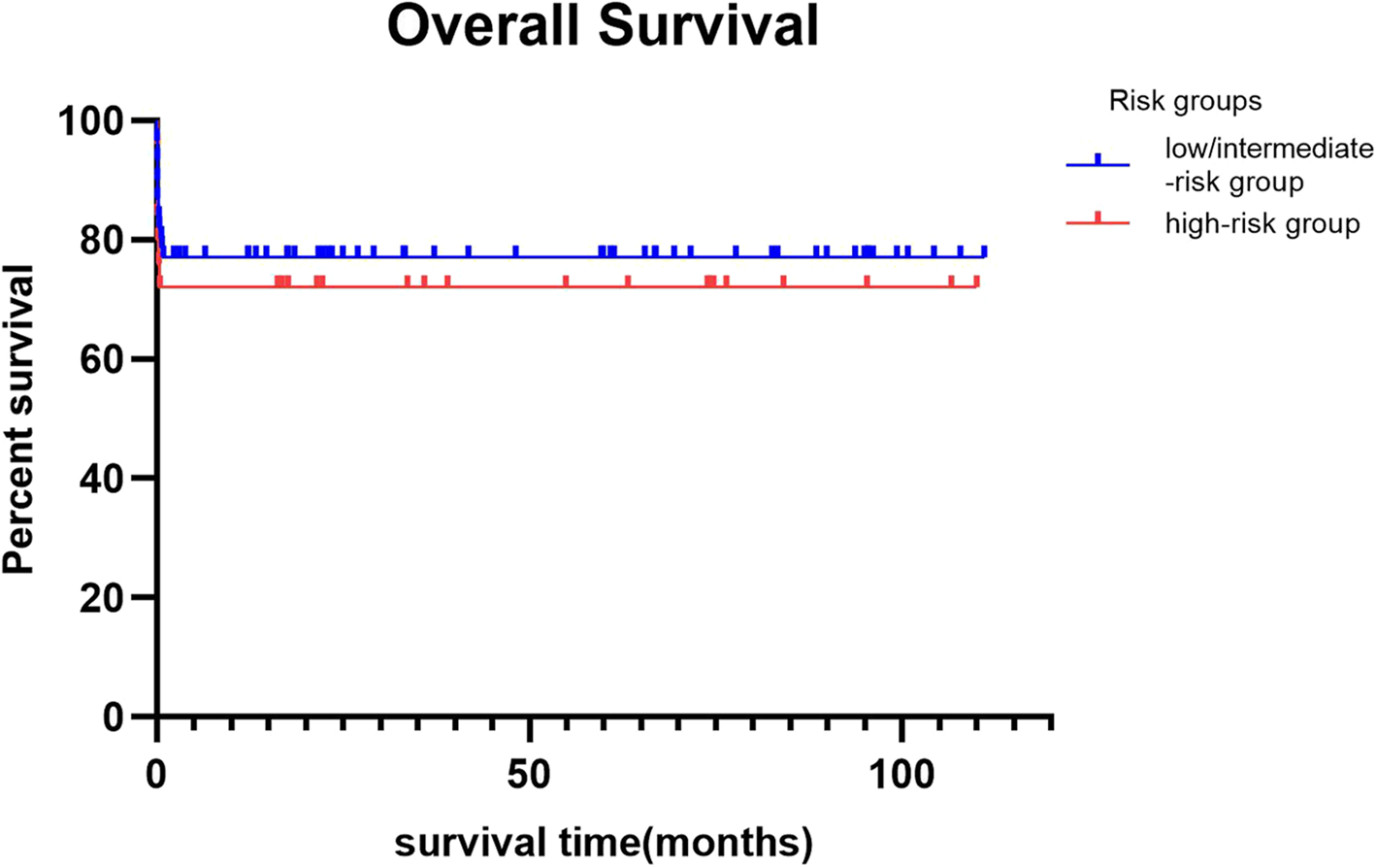
Overall survival based on risk stratification

There was no significant difference in early mortality between the low/intermediate-risk group and the high-risk group (23.0% vs. 28.0%,χ = 0.245, *P* = 0.621). There was also no significant difference in OS (χ = 0.215, *P* = 0.643, Fig. 1).

### Correlation between WBC and TNF-α

TNF-α and IL-6 were tested in 15 of the patients in this study at admission. One patient had an IL-6 level of 0.09 pg/ml and a TNF-α level of 250 pg/ml, which was an extreme value and was not included in the analysis. Fourteen patients were included in the analysis; the median TNF-α level was 8.12 (4.0–44.0) pg/ml; and the median IL-6 level was 6.61 (2.0–87.4) pg/ml.

**Fig. 2:**
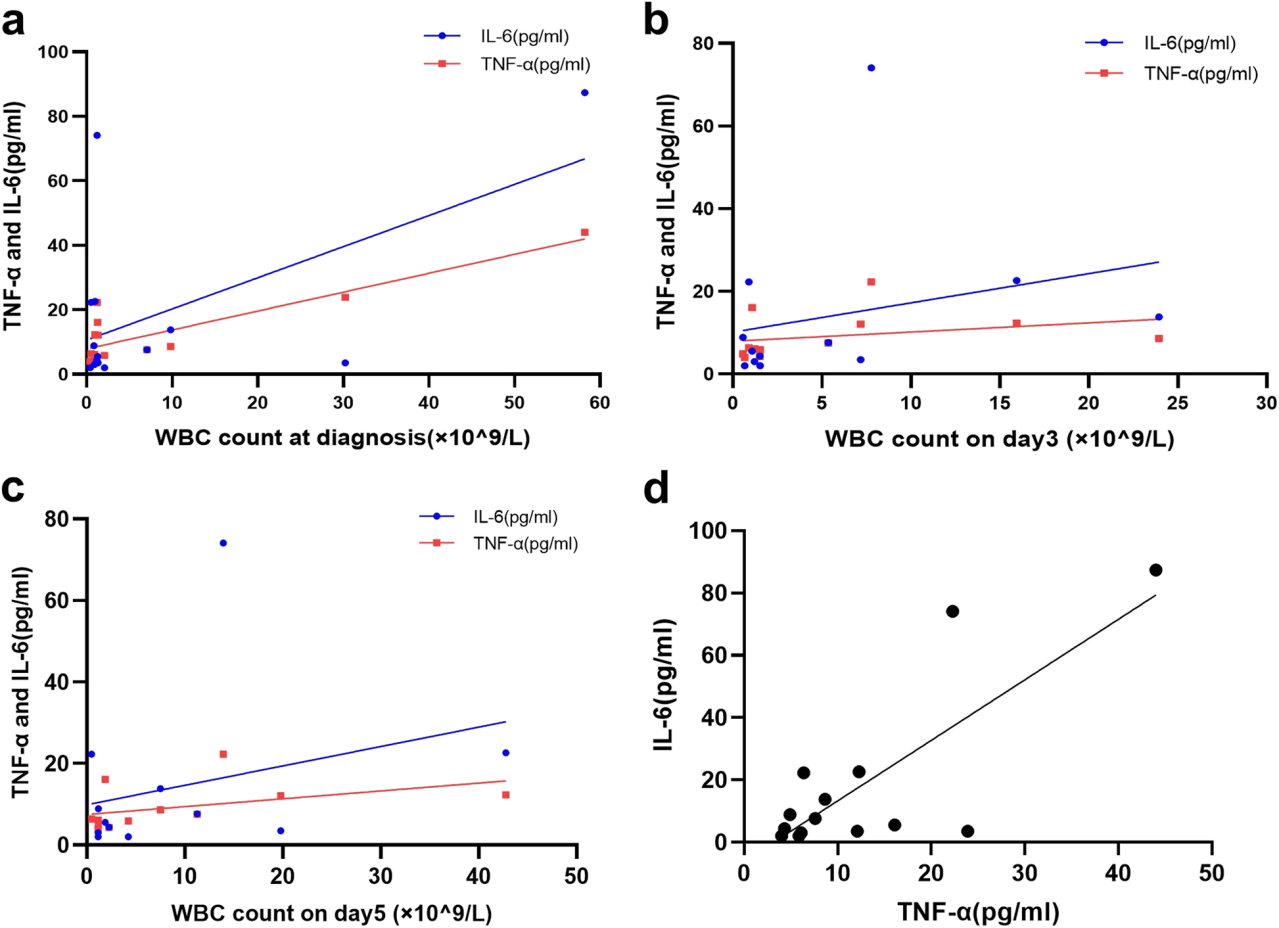
Scatter plot of TNF-α, IL-6 and WBC count. **a** scatter plot of TNF-α at diagnosis and WBC count at diagnosis; **b** scatter plot of TNF-α at diagnosis and WBC count on Day3; **c** scatter plot of TNF-α at diagnosis and WBC count on Day5; **d** scatter plot of TNF-α and IL-6 at diagnosis

The results showed that WBC count at diagnosis, WBC count on Day 3 and WBC count on Day 5 had moderate positive correlations with the TNF-α level at diagnosis, and the correlation coefficients were 0.648 (*P* = 0.012), 0.615 (*P* = 0.033), and 0.609 (*P* = 0.035), respectively. The WBC count had no correlation with IL-6. TNF-α at diagnosis had a moderate positive correlation with IL-6 (rs = 0.537, *P* = 0.048), (Fig. 2).

## Discussion

### Differentiation syndrome

Differentiation syndrome (DS) usually occurs during induction therapy in APL after the initiation of all-trans retinoic acid (ATRA) and/or arsenic trioxide (ATO). DS can be a life-threatening complication and is one of the major causes of early death [12, 13].

Leukocytosis is a major risk factor for DS [6, 14]. A previous study indicated that the WBC count was higher among patients who developed DS than among those who did not [11]. Yoon et al. [15] found that a maximum WBC count> 43 × 10^9/L correlated significantly with both early death and DS. It also revealed that the maximum WBC count was better than the WBC count at diagnosis in predicting early death and DS. A study from China showed that a peak white blood cell count after chemotherapy > 20 × 10^9/L was an independent predictor of DS [16]. Our previous study [17] also found that WBC count at chemotherapy was the only independent risk factor for the occurrence of DS. We found that a peak WBC count ≥16 × 10^9/L was an independent risk factor for the occurrence of DS, and a WBC count ≥23 × 10^9/L at chemotherapy was an independent risk factor for severe differentiation syndrome. The results of our study are basically consistent with the results of the above studies and confirms that leukocytosis is an important factor in predicting DS. In addition, although WBC count at diagnosis is an important marker of Sanz risk stratification, it is not related to the occurrence and severity of DS. Our study further indicates that the WBC count at chemotherapy may be related to the severity of DS. These results may help clinicians decide when to initiate cytotoxic chemotherapy more precisely. Although some studies believed that cytotoxic chemotherapy was not essential for APL patients with low-intermediate risk [18], we found that WBC count ≥10 × 10^9/L on Day 5 was an independent risk factor for DS. This suggests that once the WBC count is higher than 10 × 10^9/L, cytoreduction therapy should be considered to reduce the risk of DS in patients with low-intermediate risk.

In this study, we tried to find other more easily available biomarkers to predict DS. Some previous studies believed that the pathogenesis of DS was related to cytokine storms [19]. Cytokines are inflammatory mediators involved in immune reactions. Proinflammatory cytokines, including IL-1, IL-6, and TNF-α, contribute to the pathology and progression of numerous diseases, such as inflammatory diseases and cancers [20]. The levels of TNF-*α* and IL-6 were identified to be higher in APL patients than in healthy subjects [21]. ATRA induced an increase in IL-6 secretion [22]. Basic experimental studies showed that TNF-α was significantly associated with initial hyperleukocytosis, which is involved in all steps of leukemogenesis, including cellular transformation, proliferation, angiogenesis, and extramedullary infiltration [23], and TNF-α was overexpressed in differentiating APL cells [24]. Therefore, we explored the relationship between IL-6 and TNF-α levels and DS in APL during induction therapy. Our results showed that the proliferation of WBCs were closely related to TNF-α levels, but not IL-6. TNF-α may be a potential marker to predict DS risk in APL during induction treatment.

### Early death

Differences in early mortality are associated with several factors. There was a consensus on the effect of age and WBC count on early death. An analysis of 7190 adults with APL from a large National Cancer Database showed that early mortality correlated with age distribution [25]. The ED rate of elderly patients was significantly greater than that of young patients [13]. Two studies found that age > 60 years was an independent risk factor for early death [26, 27]. A higher white blood cell count was another important risk factor associated with early death [28]. A WBC count > 30 × 10^9/L during therapy was one independent factor for early death [29]. Our previous study [17] also found that WBC count at chemotherapy was the only independent risk factor for the occurrence of early death. A retrospective analysis of 368 newly diagnosed APL patients from China showed that age ≥ 50 years and WBC count ≥10 × 10^9/L were independent risk factors for early death [30]. A population-based study from China enrolled 1233 patients with newly diagnosed APL and showed that age (≥60 years) and white blood cell count (> 10 × 10^9/L) were independent risk factors for ED in the multivariate analysis [31]. One report demonstrated that age > 52 years, white blood cell count > 10 × 10^9/L, PLT count ≤10 × 10^9/L, and LDH level > 500 U/L were independent risk factors for early death [32].

Our results showed that the WBC counts on Day 3 and Day 5 were significantly higher in the early death group than in the survival group. This result is consistent with the findings of Wang H Y et al. [33]. Early mortality was significantly higher in the high WBC count (≥7 × 10^9/L on Day 3) group than in the low WBC count (< 7 × 10^9/L on Day 3) group. The multivariate analysis further identified that WBC count ≥7 × 10^9/L on Day 3 was one of the independent risk factors for early death. This indicates that the occurrence of ED could be predicted if the WBC count was subnormal. A real-world study suggested that there should be increased vigilance for monitoring ED when the patient already had subnormal or normal WBC levels [34], and this result was similar to our result. Several studies have reported that the early death rate was significantly higher in the high-risk group than in the low-intermediate risk APL group [26, 30, 35]. No significant difference was found in early mortality between the low-intermediate risk and high-risk groups in our study. The early mortality was 23.0% in the patients with low-intermediate risk, and in 50% of the patients, the WBC count was normal at death. This shows that early death is still an important problem affecting overall survival in low-intermediate risk APL. To prevent early death, cytotoxic chemotherapy may still be useful to control the WBC count even within the normal range. Our study provides evidence to initiate reduction therapy, especially for patients with normal WBC levels and who have WBC levels higher than 7 × 10^9/L on Day 3.

Men had a higher early mortality according to analysis of the SEER Database [36]. Two reports showed that females had a lower rate of one-month mortality in a univariate analysis, but this was not suggested by multivariate logistic analysis [25, 27]. Our results showed that male sex was another independent risk factor for early death. Zhou J et al. [37] reported that the haplotype [AACCG] increased the risk of male APL, whereas [CGCCG] reduced the risk of female APL by haplotype analysis. ARID5B polymorphisms contribute to the male APL risk, clinical features, and prognosis. Susceptibility to APL caused by ARID5B polymorphisms was observed only in males, suggesting that the pathogenic mechanism of ARID5B may be gender specific. The impact of gender on early death needs to be further studied. Of note, in our study, we found that an initial HGB < 65 g/L was an independent risk factor for early death. This suggests that patients with an initial HGB < 65 g/L have a high risk of early death and require more transfusions and aggressive supportive measures.

## Conclusion

However, our study had some limitations. This study was a retrospective study at a single center. Some missing laboratory parameter data also restricted the number of predictors that could be included in our multivariable model, and both overfitting and decreased power were limitations. Such limitations are common to several database studies. Since our study focuses on leukocyte proliferation, the influence of private insurance and the Charlson Comorbidity Index on outcomes were not taken into account. In addition, only a few of the patients had TNF-α and IL-6 tests performed. Thus, further studies are required to verify the relationship between leukocyte proliferation and cytokines.

In conclusion, in low-intermediate risk APL, WBC ≥ 10 × 10^9/L on Day 5 or patients with peak WBC ≥ 16 × 10^9/L suggests that cytotoxic chemotherapy may need to be initiated to reduce the risk of DS. Patients with WBC count > 7 × 10^9/L on Day 3 have a higher risk of ED. Leukocyte proliferation is associated with TNF-α rather than Interleukin-6, and TNF-α may be a potential biomarker for predicting ED.

## Supplementary Information

**Additional file 1: Supplementary Table.** Risk stratification and outcomes in acute promyelocytic leukaemia patients with and without combination chemotherapy.

## Data Availability

The data that support the findings of this study are available from the corresponding author upon reasonable request.

## Abbreviations

ATRA: All-trans retinoic acid
ATO: Arsenic trioxide
APL: Acute promyelocytic leukemia
DS: Differentiation syndrome
WBC: White blood cell
HGB: Hemoglobin
PLT: Platelet
FIB: Fibrinogen
LDH: Lactate dehydrogenase
OS: Overall survival
RBC: Red blood cell
TNF-α: Tumor necrosis factor-α
IL-6: Interleukin-6
