# Supplementary material for: Effects of peripheral blood leukocyte count and tumor necrosis factor-alpha on early death in acute promyelocytic leukemia
Source: BMC Cancer. 2023 Jan 7;23:27. doi: 10.1186/s12885-022-10499-2 (PMC9824944; doi:10.1186/s12885-022-10499-2)
Supplement: Supplementary file 1 — Additional file 1: Supplementary Table. Risk stratification and outcomes in acute promyelocytic leukaemia patients with and without combination chemotherapy. [file 12885_2022_10499_MOESM1_ESM.docx]

Supplementary Table Risk stratification and outcomes in acute promyelocytic leukaemia patients with and without combination chemotherapy.

| Groups | Low/intermediate risk (n=61) | High risk  (n=25) | ED | |
| --- | --- | --- | --- | --- |
|  |  |  | Low/intermediate risk | High risk |
| Without combination chemotherapy | 26.2%(16/61) | 0 | 56.3%(9/16) | 0 |
| Combination chemotherapy | 73.8%(45/61) | 100%(25/25) | 11.1%(5/45) | 28.0%(7/25) |
| prior to day 3 | 11.1%(5/45) | 96.0%(24/25) | 40%(2/5) | 100%(7/7) |
| prior to day 5 | 33.3%(15/45) | 96.0%(24/25) ^†^: | 100%(5/5) | 100%(7/7) |

ED, early death. ^†^:one patient delayed combination chemotherapy due to complications.
